# Relationship between LRRK2 R1628P polymorphism and Parkinson's disease in Asian populations

**DOI:** 10.18632/oncotarget.10378

**Published:** 2016-07-01

**Authors:** Hui Zhao, Zhijun Kong

**Affiliations:** ^1^ Department of General Surgery, Third Affiliated Hospital of Nantong University, Wuxi, China; ^2^ Department of General Surgery, Affiliated Hospital of Nanjing Medical University, Changzhou Second People's Hospital, Changzhou, China

**Keywords:** LRRK2, polymorphism, Parkinson's disease, meta-analysis, R1628P, Gerotarget

## Abstract

Although the leucine-rich repeat kinase 2 (*LRRK2*) R1628P polymorphism has been associated with the risk of Parkinson's disease (PD) in Taiwan, China, and Singapore, there are conflicting findings regarding this relationship. Thus, the aim of the present meta-analysis was to evaluate the associations between the *LRRK2* R1628P polymorphism (rs33949390) and PD in Asian populations. A search for eligible studies was performed in PubMed, Embase, SinoMed, and the China Knowledge Resource Integrated Database, and pooled odds ratios and 95% confidence intervals were used to evaluate the strength of the association between the R1628P polymorphism and PD. This meta-analysis assessed 19 studies from 14 papers that involved a total of 9,927 PD patients and 8,602 controls and found that the R1628P polymorphism was significantly associated with the risk of PD in Asian populations. Moreover, stratification analyses indicated that the R1628P polymorphism was significantly associated with an increased risk of PD among Chinese as well as non-Chinese Asian populations and an increased risk of PD in Chinese patients from China, Taiwan, and Singapore. In a stratified analysis conducted according to age, significant associations were found for both late-onset PD and early-onset PD. The present data indicate that the R1628P polymorphism of the *LRRK2* gene contributes to PD susceptibility in Asian, especially Chinese, populations.

## INTRODUCTION

Parkinson's disease (PD), which is one of the most common neurodegenerative disorders, is characterized by tremors, rigidity, bradykinesia, and postural instability [1]. It has been estimated that approximately 9.3 million individuals older than 50 years of age around the world will suffer from PD by 2030 [2]. Although the etiology of PD remains poorly understood, genetic variations likely play important roles in its development [3]. For example, mutations in the leucine-rich repeat kinase 2 (LRRK2) gene lead to autosomal dominant PD [4, 5]. Studies have demonstrated that polymorphisms of the *LRRK2* gene are related to an increased presence of PD compared with control groups [6].

Recently, a study from three independent centers in Taiwan and Singapore demonstrated that *LRRK2* R1628P (rs33949390) polymorphism was a genetic risk factor for PD [7]. Subsequent studies have further investigated the association between the R1628P polymorphism and the risk of PD but produced conflicting results [8–20]. However, these discrepancies may be due inadequate statistical power, different ethnic populations, and small sample sizes. To overcome these limitations and to resolve the inconsistencies among previous studies, we conducted this meta-analysis to investigate whether the *LRRK2* R1628P polymorphism is associated with susceptibility to PD.

## RESULTS

### Characteristics of the published studies

**Figure 1:**
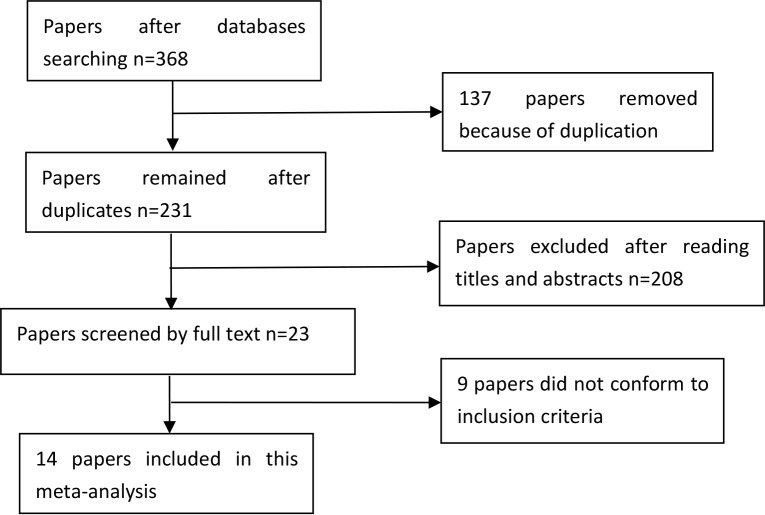
Selection for eligible papers included in this meta-analysis

A total of 368 papers were yielded after our initial search. After removing duplicates and screening the titles and abstracts, 345 papers were removed. Therefore, 23 papers were selected for further full text review. Four papers were not case-control studies [21–24]; one was a review [25]; four did not provide detailed genotype data [26–29]. Finally, we identified fourteen eligible papers [7–20] including nineteen studies in this meta-analysis. Selection for eligible papers included in this meta-analysis was presented in Figure 1. The characteristics of these included papers are summarized in Table 1. These papers were published from 2008 to 2014. All included studies conformed to HWE. The NOS scores of all included studies ranged from 5 to 7 stars, indicating that they were studies of high methodological quality. Seven studies were carried out in China [8, 9, 14, 17–20], three in Singapore [7, 19], and six in Taiwan [7, 11–13, 19]; the remaining three studies were carried out in Malaysia [15], Thailand [10] and South Korea [16].

**Table 1:** Characteristics of included studies

Author and year	Country	Case			Control			HWE	Genotypes Methods	NOS
		GG	GC	CC	GG	GC	CC			
Gopalai2014	Malaysia	659	35	1	489	18	0	0.684	PCR	6
Wu2013	Taiwan	539	34	0	481	22	0	0.616	PCR	6
Cai2013	China	482	28	0	532	18	0	0.696	PCR–RFLP	6
Wu-Chou2013	Taiwan	689	56	2	443	18	0	0.669	TaqMan	6
Fu2013	China	399	47	0	382	21	0	0.591	PCR	5
Wang2012	China	1949	63	1	1940	31	0	0.725	PCR	7
Zhou2012	China	200	2	0	207	5	0	0.862	PCR	6
Pulkes2011	Thailand	139	14	1	151	5	0	0.834	Na	6
Tan2010	Singapore	226	24	0	235	15	0	0.625	MALDI-TOF	6
Tan2010	Singapore	163	28	1	173	18	1	0.484	MALDI-TOF	6
Tan2010	Taiwan	270	23	0	281	18	0	0.592	MALDI-TOF	6
Tan2010	China	579	48	1	500	10	0	0.823	MALDI-TOF	5
Kim2010	South Korea	380	3	0	378	1	0	0.980	PCR	7
Yu2009	China	311	17	0	294	6	0	0.861	PCR-RFLP	5
Zhang2009	China	557	40	3	448	11	0	0.795	PCR	6
Ross2008	Taiwan	452	31	1	330	11	0	0.762	RFLP	6
Ross2008	Taiwan	324	21	0	302	14	0	0.687	RFLP	6
Ross2008	Singapore	237	13	0	244	6	0	0.848	RFLP	6
Lu2008	Taiwan	772	60	2	523	20	0	0.662	PCR	6

HWE, Hardy–Weinberg equilibrium; NOS, Newcastle-Ottawa Scale; Na, not available

**Figure 2:**
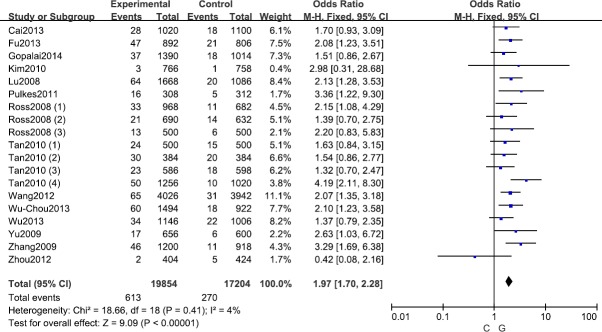
Forest plot shows odds ratio for the associations between *LRRK2* R1628P polymorphism and PD risk (C *vs* G)

### Meta-analysis: LRRK2 R1628P polymorphism (rs33949390)

**Figure 3:**
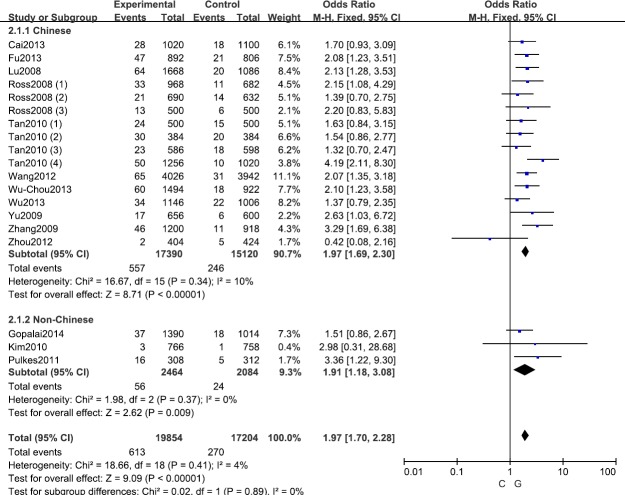
Stratification analyses of ethnicity between *LRRK2* R1628P polymorphism and PD risk (C *vs* G)

**Figure 4:**
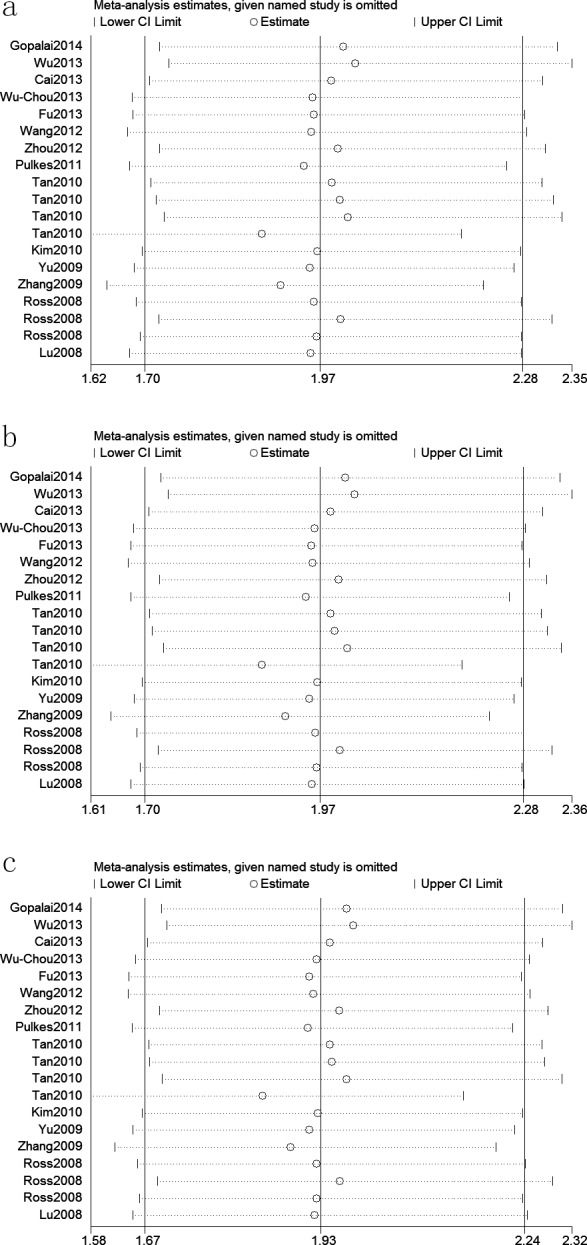
Sensitivity analyses between *LRRK2* R1628P polymorphism and PD risk (C *vs* G; b: CC+GC *vs*. GG; c: GC *vs*. GG)

**Figure 5:**
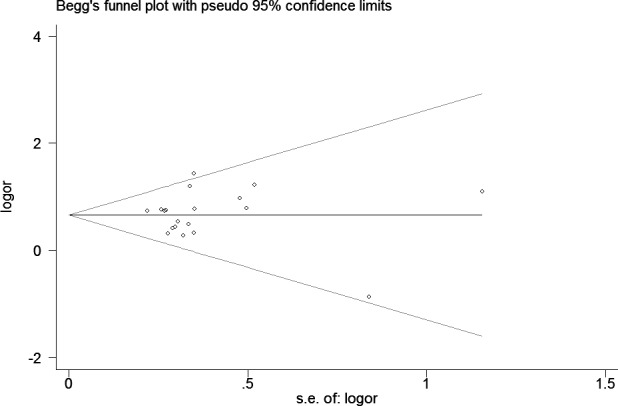
Begg's tests for publication bias between *LRRK2* R1628P polymorphism and PD risk (C *vs* G)

A fixed effects model was conducted for allele model (C *vs*. G), dominant model (CC + CG *vs*. GG) and heterozygous model (CG *vs*. GG) without significant heterogeneity. Due to the deficiency of CC genotypes in included studies, we could not calculate the data of recessive model and homozygous model. As shown in Table 2, R1628P polymorphism was significantly associated with PD risk (C *vs*. G: OR, 1.97; 95% CI, 1.70-2.28, *P* < 0.00001, Figure 2; CC+GC *vs*. GG: OR, 1.97; 95% CI, 1.70-2.28, *P* < 0.00001; GC *vs*. GG: OR, 1.93; 95% CI, 1.67-2.24, *P* < 0.00001). Stratification analyses were conducted according to ethnicity, age and country (Table 2). The results indicated that R1628P polymorphism was significantly associated with the increased risk of PD among Chinese as well as non-Chinese (C *vs*. G, Figure 3). Furthermore, we found that R1628P polymorphism might increase the risk of PD patients from China, Taiwan, and Singapore. In the stratified analysis by age, we also found that R1628P polymorphism was associated with PD risk. We assessed sensitivity by omitting each study once at a time in every genetic model. The pooled ORs for the effect of R1628P polymorphism on the risk for PD indicated that our data were stable and trustworthy (Figure 4). Both Egger's and Begg's tests were used to evaluate the publication bias of this meta-analysis. The results revealed that there was no obvious publication bias in overall analysis for R1628P polymorphism (C *vs*. G, *P*_begg_ = 0.753 and *P*_egger_ = 0.964, Figure 5; CC + CG *vs*. GG, *P*_begg_ = 0.649 and *P*_egger_ = 0.987; CG *vs*. GG, *P*_begg_ = 0.552 and *P*_egger_ = 0.984).

**Table 2:** Meta-analysis of associations between the R1628P polymorphism and PD risk

Comparison	Overall and Stratification analyses	Studies	OR (95% CI)	*P*-value	P for heterogeneity	I2 (%)
C vs. G	Overall	19	1.97(1.70,2.28)	<0.00001	0.41	4.0
	Chinese	16	1.97(1.69,2.30)	<0.00001	0.34	10.0
	Non-Chinese	3	1.91( 1.18,3.08)	0.009	0.37	0.0
	Chinese in China	7	2.33(1.84,2.94)	<0.00001	0.14	38.0
	Chinese in Taiwan	6	1.75(1.38,2.22)	<0.00001	0.64	0.0
	Chinese in Singapore	3	1.67 (1.12,2.49)	0.01	0.83	0.0
	EOPD	3	2.00(1.05,3.83)	0.04	0.68	0.0
	LOPD	3	2.48 (1.24,4.95)	0.01	0.09	59.0
CC+GC vs. GG	Overall	19	1.97(1.70,2.28)	<0.00001	0.49	0.0
	Chinese	16	1.98(1.69,2.31)	<0.00001	0.41	4.0
	Non-Chinese	3	1.86( 1.14,3.03)	0.01	0.39	0.0
	Chinese in China	7	2.33(1.84,2.94)	<0.00001	0.16	36.0
	Chinese in Taiwan	6	1.74(1.37,2.21)	<0.00001	0.70	0.0
	Chinese in Singapore	3	1.73 (1.15,2.62)	0.009	0.86	0.0
	EOPD	3	2.03(1.05,3.90)	0.03	0.67	0.0
	LOPD	3	2.45(1.26,4.74)	0.008	0.12	54.0
GC vs. GG	Overall	19	1.93(1.67,2.24)	<0.00001	0.57	0.0
	Chinese	16	1.99(1.68,2.36)	<0.00001	0.62	0.0
	Non-Chinese	3	1.79 (1.09,2.93)	0.02	0.43	0.0
	Chinese in China	7	2.28(1.80,2.89)	<0.00001	0.19	32.0
	Chinese in Taiwan	6	1.71(1.34,2.17)	<0.0001	0.77	0.0
	Chinese in Singapore	3	1.75(1.16,2.65)	0.008	0.87	0.0
	EOPD	3	2.06(1.09,3.92)	0.03	0.67	0.0
	LOPD	3	2.37(1.54,3.66)	<0.0001	0.16	46.0

EOPD, early-onset Parkinson's disease; LOPD, late-onset Parkinson's disease; OR, odds ratio; 95% CI, 95% confidence interval.

## DISCUSSION

PD is a progressive neurodegenerative disease, which is caused primarily by the loss of dopaminergic neurons in the substantia nigra compacta and the presence of intracellular inclusions known as Lewy bodies. Although the potential etiology of PD remains poorly understood, PD is thought to be caused by both genetic and environmental factors. To date, several candidate genes including *LRRK2* have been reported to be associated with the risk of PD [30]. Many studies have demonstrated that genetic risk factors play a crucial role in the susceptibility to PD, and recently, the role of *LRRK2* gene variants in the development of PD have sparked a great deal of interest [31]. The *LRRK2* gene encodes a large multidomain protein that includes an ankyrin repeat (ANK), a leucine-rich repeat (LRR), Ras of complex proteins (ROC; GTPase), C-terminal of ROC (COR), mitogen-activated kinase kinase kinase (MAPKKK), and WD40 domains [32, 33]. The R1628P polymorphism is located in the COR region. Some studies shown that the R1628P may affect the interaction between the different functional domains of LRRK2 or its interaction with other proteins, which in turn, may result in altered LRRK2 protein function [29].

Many studies investigated the relationship between the *LRRK2* R1628P polymorphism and the risk of PD in Asian individuals [7–20], but produced contradictory results. Therefore, it was necessary to conduct a meta-analysis to determine whether this polymorphism is associated with PD susceptibility in Asian populations. The findings of the present meta-analysis suggested that *LRRK2* R1628P polymorphism was associated with an increased risk of PD among Asian populations. Previous meta-analyses [26, 34] investigating the R1628P polymorphism in terms of PD susceptibility produced conflicting findings. Ross et al. [26] analyzed 1,376 PD cases and 962 controls and found that R1628P is not associated with a risk of PD in Asian populations, while Wu et al. [34] determined that this polymorphism actually increases the risk of PD in Asians, which is consistent with the present findings. The findings of the present meta-analysis and Wu et al. are likely more robust than those of Ross et al. due to the use of larger sample sizes.

The stratification analyses in this meta-analysis also indicated that the R1628P polymorphism is significantly associated with increased risks of PD among Chinese and non-Chinese individuals, which was not found by Wu et al. [34]. However, the associations among the SNPs in genes associated with disorders were greatly affected by the number of subjects in the study. The results of this meta-analysis of non-Chinese individuals should be interpreted with caution because only three of the analyzed studies included non-Chinese subjects (1,232 PD cases and 1,402 controls). Furthermore, two studies investigating Japanese subjects suggested that the R1628P variant is not associated with susceptibility to PD [7, 27]. Tan et al. also demonstrated that R1628P was not a genetic risk factor for Malaysian and Indian populations [28]. However, these three studies did not provide detailed genotype data, and thus, it is not advisable to exclude the possibility that the R1628P polymorphism is not related to the risk of PD in non-Chinese ethnicities if the present meta-analysis had included these data [7, 27, 28]. Therefore, it is reasonable to assume that the *LRRK2* R1628P polymorphism may be a specific risk factor of PD among Chinese individuals, which suggests the need for future larger-scale studies to validate this assumption. The stratified analysis in the present meta-analysis also indicated that the R1628P polymorphism increases the risk of PD in Chinese patients from China, Taiwan, and Singapore. In the stratified analysis that was conducted according to age, significant associations were found in both late-onset and early-onset PD cases, but this finding should be interpreted with caution due to limited sample sizes. A sensitivity analysis indicated that the present data were stable and trustworthy.

The present meta-analysis has several limitations that should be should be taken into consideration. First, this study did not investigate the association between the *LRRK2* R1628P polymorphism and PD in Caucasian populations. Second, unpublished trials may have been excluded unintentionally and could have resulted in a publication bias; however, there was no evidence of this. Third, the present findings were based on unadjusted estimates that did not consider confounding factors such as age, sex, or environmental factors, and thus, the analysis could have been more precise if individual data were available. Fourth, the results of the stratified analyses should be interpreted with caution due to limited sample sizes. Future larger-scale studies are urgently needed to confirm these results. Fifth, it is impossible to include all the languages when we performed the literature search.

In conclusion, this meta-analysis indicates that *LRRK2* R1628P polymorphism is significantly associated with an increased risk of PD in Asians, especially among Chinese subjects. Future large-scale studies are necessary to characterize the association between the *LRRK2* R1628P polymorphism and PD.

## MATERIALS AND METHODS

### Literature search

We performed a comprehensive search in PubMed, Embase, SinoMed, and the China Knowledge Resource Integrated Database to identify studies through March 1, 2016 that were related with the *LRRK2* gene polymorphism and PD. The following search terms were used: “Leucine-rich repeat kinase 2,” “LRRK2,” “PARK8,” “Parkinson's disease,” “Parkinson disease,” “PD,”“SNP,” and “polymorphism”. Two independent investigators conducted the search. No language or other restrictions were placed on the search. We also performed a manual search of references cited in published papers to identify other initially omitted studies. Any disagreements were resolved by consensus. Criteria for the inclusion in this analysis were: (1) studies that evaluated the association between *LRRK2* R1628P polymorphism (rs33949390) and PD in Asian populations, (2) studied on human beings, (3) study provided sufficient data to calculate the Odds ratios (ORs) and 95% confidence intervals (CIs), and *P* value, and (4) case-control study. Exclusion criteria were: (1) duplication of previous publications; (2) review, editorial, or other non-original studies; (3) studies without detailed genotype data; (4) studies without controls.

### Data extraction and quality assessment

For all eligible studies, the extracted information including: name of first author, publication year, country of origin, and *LRRK2* R1628P genotype frequency in cases and controls. Extraction of the data was independently performed by two authors according to the inclusion criteria. All included studies were evaluated using the

Newcastle-Ottawa Scale (NOS) [35]. They compared results and agreed on a consensus; disagreements were resolved by discussion.

### Statistical analysis

All statistical analyses were performed using the Stata 11.0 software (StataCorp, College Station, TX, USA) and RevMan 5.3 (The Nordic Cochrane Centre, Copenhagen, Denmark). Pooled ORs and 95% CIs were calculated to evaluate the strength of the association between *LRRK2* R1628P polymorphism (rs33949390) and PD. Heterogeneity was evaluated by the Q statistic (significant at *P* < 0.1) and I^2^ statistic (where > 50% indicates significant heterogeneity) [36]. A fixed-effect model was used for comparing the trials without showing heterogeneity, whereas a random effect model was selected for comparing trials showing heterogeneity. Pooled ORs were calculated for allele model, dominant model, recessive model, homozygous model, and heterozygous model. We performed sensitivity analyses by omitting each study in turn to determine the effect on the test of heterogeneity and evaluated the stability of the overall results. Hardy-Weinberg equilibrium (HWE) was assessed in the controls using Pearson's χ^2^ test. Potential publication bias was investigated with the use of Begger's and Egger's linear regression test [37]. A *P* value < 0.05 was considered to indicate statistically significant.

## Abbreviations

CI: confidence interval
OR: odds ratio
PD: Parkinson's disease
LRRK2: Leucine-rich repeat kinase 2
NOS: Newcastle-Ottawa Scale
HWE: Hardy-Weinberg equilibrium
